# Complete Genome Sequence of *Bacillus thuringiensis* Strain 407 Cry-

**DOI:** 10.1128/genomeA.00158-12

**Published:** 2013-01-31

**Authors:** Anna E. Sheppard, Anja Poehlein, Philip Rosenstiel, Heiko Liesegang, Hinrich Schulenburg

**Affiliations:** a Department of Evolutionary Ecology and Genetics, Zoological Institute, Christian-Albrechts University of Kiel, Kiel, Germany; b Goettingen Genomics Laboratory, Institute of Microbiology and Genetics, Georg-August-University of Goettingen, Goettingen, Germany; c Institute of Clinical Molecular Biology, Christian-Albrechts University of Kiel, Kiel, Germany

## Abstract

*Bacillus thuringiensis* is an insect pathogen that has been used widely as a biopesticide. Here, we report the genome sequence of strain 407 Cry-, which is used to study the genetic determinants of pathogenicity. The genome consists of a 5.5-Mb chromosome and nine plasmids, including a novel 502-kb megaplasmid.

## GENOME ANNOUNCEMENT

*Bacillus thuringiensis* is a Gram-positive bacterium that is pathogenic towards a range of insect and nematode species. This is largely mediated through the production of crystal (cry) toxin proteins, which vary among *B. thuringiensis* strains and enable the infection of particular hosts. Because of the insecticidal activities of the cry toxins, *B. thuringiensis* has been used widely as a biopesticide, and there is a great deal of interest to understand further its pathogenic properties and how host resistance may evolve.

Strains of *B. thuringiensis* vary in their amenability to genetic manipulation, and acrystalliferous strains may have higher transformation frequencies than do crystal-producing strains (1). *B. thuringiensis* strain 407 was isolated as a lepidopteran-active strain, and an acrystalliferous derivative, 407 Cry-, was produced through culturing at a high temperature (2). The 407 Cry- strain can be transformed easily and it can be used to perform targeted gene knockouts; therefore, the strain has been used successfully as a genetic model for studying virulence mechanisms and the pathways that contribute to pathogenicity (3–7). So far, the complete genome sequences of five *B. thuringiensis* strains have been reported (1, 8–11). Here, we report the complete genome sequence of *B. thuringiensis* strain 407 Cry-, which will assist in the use of this strain as a genetic model, as well as will contribute to studies of genome structure and function in *B. thuringiensis*.

Genomic DNA was isolated from 407 Cry- using a DNeasy blood and tissue kit (Qiagen). Whole genome sequencing was performed using the Roche 454 Genome Sequencer FLX platform. A total of 478,069 single-end reads with an average length of 387 bases were assembled using Genome Sequencer (GS) *de novo* Assembler version 2.6 (Roche), which generated a total of 189 contigs. Repeats were resolved and gaps between contigs were closed using PCR and Sanger sequencing. The copy number of each plasmid was estimated based on read coverage relative to the chromosome. Annotation was performed initially by the Integrated Microbial Genomes (IMG) annotation pipeline (12) and was curated using Swiss-Prot/trEMBL BLAST comparisons.

The genome of 407 Cry- consists of a 5.5-Mb chromosome and nine plasmids, named BTB_2p, BTB_5p, BTB_6p, BTB_7p, BTB_8p, BTB_9p, BTB_15p, BTB_78p, and BTB_502p; plasmid sizes range from 2,062 bp to 501,911 bp. BTB_502p is the largest *B. thuringiensis* plasmid reported to date and is entirely novel, with BLASTn searches revealing no similarity to the published sequences for >95% of the plasmid sequence. The copy numbers of the plasmids were estimated to be 16, 10, 8.8, 14, 11.5, 26, 3.4, 1.6, and 1.8 copies per cell, respectively. The G+C content of the chromosome is 35.4% and those of the plasmids range from 29.7% to 35.7%. The total number of predicted genes is 6,635, with 5,714 genes located on the chromosome and 921 genes on the plasmids.

### Nucleotide sequence accession numbers. 

The sequence of the *B. thuringiensis* 407 Cry- chromosome has been deposited in GenBank under the accession no. CP003889 and the plasmids under the accession no. CP003890 to CP003898.
